# Steps towards Smarter Solutions in Optometry and Ophthalmology—Inter-Device Agreement of Subjective Methods to Assess the Refractive Errors of the Eye

**DOI:** 10.3390/healthcare4030041

**Published:** 2016-07-13

**Authors:** Arne Ohlendorf, Alexander Leube, Siegfried Wahl

**Affiliations:** 1 Institute for Ophthalmic Research, University of Tuebingen, Geschwister-Scholl-Platz, 72074 Tübingen, Germany; alexander.leube@uni-tuebingen.de (A.L.); siegfried.wahl@uni.tuebingen.de (S.W.); 2 Carl Zeiss Vision International GmbH, 73430 Aalen, Germany

**Keywords:** public health, optometry, subjective refraction, refractive errors, agreement

## Abstract

Purpose: To investigate the inter-device agreement and mean differences between a newly developed digital phoropter and the two standard methods (trial frame and manual phoropter). Methods: Refractive errors of two groups of participants were measured by two examiners (examiner 1 (E1): 36 subjects; examiner 2 (E2): 38 subjects). Refractive errors were assessed using a trial frame, a manual phoropter and a digital phoropter. Inter-device agreement regarding the measurement of refractive errors was analyzed for differences in terms of the power vector components (spherical equivalent (SE) and the cylindrical power vector components J0 and J45) between the used methods. Intraclass correlation coefficients (ICC’s) were calculated to evaluate correlations between the used methods. Results: Analyzing the variances between the three methods for SE, J0 and J45 using a two-way ANOVA showed no significant differences between the methods (SE: *p* = 0.13, J0: *p* = 0.58 and J45: *p* = 0.96) for examiner 1 and for examiner 2 (SE: *p* = 0.88, J0: *p* = 0.95 and J45: *p* = 1). Mean differences and ±95% Limits of Agreement for each pair of inter-device agreement regarding the SE for both examiners were as follows: Trial frame vs. digital phoropter: +0.10 D ± 0.56 D (E1) and +0.19 D ± 0.60 D (E2), manual phoropter vs. trial frame: −0.04 D ± 0.59 D (E1) and −0.12 D ± 0.49 D (E2) and for manual vs. digital phoropter: +0.06 D ± 0.65 D (E1) and +0.08 D ± 0.45 D (E2). ICCs revealed high correlations between all methods for both examiner (*p* < 0.001). The time to assess the subjective refraction was significantly smaller with the digital phoropter (examiner 1: *p* < 0.001; examiner 2: *p* < 0.001). Conclusion: “All used subjective methods show a good agreement between each other terms of ICC (>0.9). Assessing refractive errors using different subjective methods, results in similar mean differences and 95% limits of agreement, when compared to those reported in studies comparing subjective refraction non-cylcoplegic retinoscopy or autorefraction”.

## 1. Background

Uncorrected refractive errors, such as myopia, hyperopia and astigmatism, have a high impact on the prevalence of visual impairment or blindness, as recently reviewed by Naidoo and colleagues [1]. Since it is known that the prevalence of refractive errors is increasing [2], its assessment will become one of the major tasks in the public health sector worldwide. Digitalization is already affecting our lives, and its influence will increase in the future; the aim is to develop smart products that are able to assess refractive errors in order to provide adequate correction for people living in developing, as well as in industrial, countries. Several “smart” solutions are already available that assess refractive errors objectively and subjectively. For example, the company EyeNetra (EyeNetra Inc., Somerville, MA, USA) developed a smartphone-based refraction for mobile measurements of refractive errors [3,4,5] that uses a pinhole optic to display a stripe pattern on the participant’s retina, where the task of the subject is to align a red and green stimulus [3]. Another smartphone-based autorefractor is SVOne (Smart Vision Labs, New York, NY, USA), where a portable Hartmann-Shack wavefront aberrometer is attached to a smartphone [6]. In contradiction, Opternative (Opternative Inc., Chicago, IL, USA) is an online solution that aims to measure the refraction of the eye in a self-directed way, using a computer-based response to presented stimuli (https://www.opternative.com/)  [7]. Thus far, the performance of these products is limited. Using Opternative, the range of refractive errors that can be measured is between 0 D and −4 D [7]. EyeNetra claims to have an extended range of refractive errors that can be measured (−12.5 D to +5.5 D) [8]. While the assessment of the refractive errors is the core competence of eye care professionals (ECPs), all used methods have to agree between each other and have to be reproducible, as well as repeatable. Published data on the variability of refraction, either assessed subjectively or objectively, were mainly evaluated during studies that assessed the variability and repeatability of auto-refractors. Most of the studies [9,10,11] used two repetitive measurements of subjective refraction per subject, only Rosenfield and Chiu, 1995 [12] assessed the subjective refraction with five repeated measurements. The reported 95% limit of agreement for the subjective measurement of the spherical equivalent refractive error, which was ±0.29 D, suggesting that the subjective refraction is accurate to about a quarter diopter [12]. In contradiction, Zadnik, Mutti and Adams, 1992 [10] reported a 95% limit of agreement for the SE of ±0.63 D for cycloplegic and of ±0.72 D for non-cycloplegic refraction. Studies regarding the agreement between the earlier mentioned “smart” products, such as EyeNetra or SVOne, with the traditional methods, showed good agreement between traditional and smartphone-based technologies. Clinical results in 27 subjects (54 eyes) with refractive errors ranging from 0 D to −6 D for the EyeNetra (Netra G) device showed that the difference between the EyeNetra and the subjective refraction for spherical equivalent error SE was 0.31 ± 0.37 D [4]. Later, it was shown that two different versions of the EyeNetra device overestimated myopia (spherical error) by 0.48 ± 0.66 D (Netra G #243) in 24 subjects and by 0.64 ± 0.71 D (Netra G #244 with a smaller pupillary distance) in 19 subjects [3]. In 50 visually-normal, young subjects with an average spherical equivalent refractive error of −2.87 D, the 95% limit of agreement for the assessment of the power vector components (J0 and J45) was highest for the SVOne device, when compared to retinoscopy and two autorefractors (Topcon KR-1W and Righton Retinomax-3) under cycloplegic and non-cylcoplegic conditions [6]. In case of the SE, no significant differences were found between the different methods and devices and the 95% limit of agreement for the SVOne was comparable to Retinoscopy under non-cycloplegic conditions [6]. Clinical data for 60 eyes from 30 subjects (aged between 18 and 40 years and with spherical refractive errors of up to −4 D and astigmatism of up to −2 D) for the Opternative software are available on their webpage [7], but is not yet published. In summary, they report that a difference in the spherical equivalent difference of 0.25 diopter was apparent in 70% of the eyes, and a spherical equivalent difference of 0.50 diopter or less was found in 90% of the eyes. Nevertheless, in their review, Goss and Grosvenor, 1996 [13] concluded that the assessment of refractive errors using subjective methods, such as a trial frame or a phoropter, are far better compared to any other method, and that the agreement of the measurement of the SE (either intra-examiner or inter-examiner) was close to ±0.25 D in 80% of the measurements, while the agreement was ±0.50 D for 95% of the measurements for the SE, the sphere and the cylinder power.

The purpose of the current study was to investigate the inter-device agreement and mean differences between a newly developed digital phoropter and the two standard methods (trial frame and manual phoropter) currently used to assess the refractive error of the eye.

## 2. Methods

### 2.1. Subjects

Inclusion criterion for participation was a refractive error of less than ±8.0 D of spherical ametropia, ≤−4.0 D of astigmatism and best corrected visual acuity of minimum 0.0 logMAR. Subjects with known ocular diseases were not allowed to participate in the course of the study. Two examiners (examiner 1: Author AO and examiner 2: Author AL, both certified optometrists) measured the refractive errors in two independent studies, using the same devices/methods but different subjects. Examiner 1 measured refractive errors in 36 subjects, aged 23–47 years (mean: 36.4 ± 7.4 years), with a mean spherical refractive error (S) of −0.71 ± 1.62 D (range +2 D to −5.75 D and a mean astigmatic refractive error of −0.59 ± 0.45 D (range 0 D to −2 D)). The study group for examiner 2 included 38 subjects, aged 22–47 years (mean: 36.7 ± 7.1 years), with a mean S of −0.83 ± 1.39 D (range +1.75 to −3.5 D and an average astigmatic error of −0.67 ± 0.49 D (range 0 D to −2 D)). All subjects were naïve to the purpose of the experiment. The study course was approved from the Ethics Commission of the Medical Faculty of the University of Tuebingen. The research followed the tenets of the Declaration of Helsinki, informed consent was obtained from all subjects after explanation of the nature and possible consequences of the study.

### 2.2. Equipment

To assess the refractive errors of an individual’s eye objectively, a wavefront-based autorefractor was used (ZEISS i.Profiler plus, Carl Zeiss Vision GmbH, Aalen, Germany). Subjective refraction was assessed using a Subjective Refraction Unit (SRU), a trial frame (UB4, Oculus, Wetzlar, Germany) in combination with trial lenses (BK1, Oculus, Wetzlar, Germany) and a manual phoropter (American Optical Phoropter M/N 11320, American Optics, Buffalo, NY, USA). The SRU includes a digital phoropter (ZEISS Visuphor 500, Carl Zeiss Vision GmbH, Aalen, Germany) and a screen to display optotypes (ZEISS Visuscreen 500). The digital phoropter covers spherical refractive errors from −19 D to +16.75 D and astigmatic errors from 0 D to ±8.75 D. A Tablet PC (iPad3, Apple, Cupertinoj, CA, USA) was used that controlled the mentioned devices from the SRU, using an application called i.Com mobile (Carl Zeiss Vision GmbH). All optotypes (SLOAN Letters) to subjectively measure the refractive errors were displayed on a digital visual acuity chart (ZEISS Visuscreen 500, Carl Zeiss Vision GmbH) at a distance of 6 m with a minimum luminance of 250 cd/m^2^.

### 2.3. Experimental Procedures

Objective measurements of refractive errors were obtained three times for each eye prior to the subjective measurements using a wavefront-based autorefractor (ZEISS i.Profiler plus) and the most positive reading served as the starting value for the subjective refraction (sphere, cylinder, axis). Both examiners measured the refractive errors under monocular as well as binocular conditions. The procedure was as follows: Objective refraction was measured prior to the subjective refraction by a technician in order to test if the subject meets the exclusion and inclusion criteria and both examiners were masked to the results of the autorefraction measurement. Following, the examiners conducted the subjective refraction, starting either with the manual phoropter or the digital phoropter (the procedure was randomized), since it was possible to mask the “workflow” (the power of the used lenses and the results) from the examiner. In case of the manual, as well as the digital, phoropter, both examiners were masked from the results of the subjective refraction. The trial frame refraction was performed at the end of the study. The SRU provides a preconfigured refraction workflow that guides the eye care professional through the process of the subjective refraction procedure. This workflow contains the following steps to determine the monocular refractive error, starting with the right eye: (a) determination of the best sphere; (b) determination of a cylindrical error, using a Jackson cross cylinder (if a cylindrical error was measured using the objective method, this step is skipped); (c) determination of the axis of the existing cylindrical refractive error; (d) determination of the power of the existing cylindrical refractive error; (e) fine adjustment of the sphere (monocular). The same workflow is used to assess the subjective refraction of the left eye, after measurements of the right eye are finished. After assessment of the monocular prescription, the following binocular tests are done: (a) polarized duochrome test to achieve binocular balance and (b) polarized optotypes for the assessment of the best sphere under binocular conditions. Lighting conditions during the experiments followed the international standards DIN EN ISO 8596 and 8597, which defines an ambient luminance of 80–320 cd/m^2^.

### 2.4. Analysis

Both examiners used three methods of subjective refraction to assess the refractive errors in two separate study cohorts. Examiner 1 measured refractive errors in a group of 36 subjects, while examiner 2 assessed the refractive errors in a group of 38 subjects. Data about the refractive errors of the left eyes after the monocular refraction was used to assess the inter-device agreement between the different methods. Refractive errors were analyzed for the power vector components spherical equivalent refractive error (SE) and J0 and J45 that were introduced by Thibos, Wheeler and Horner, 1997 [14]. Time needed for each measurement of refraction including the tests under binocular conditions, was directly saved by the i.Com software. The time durations for each used method were analyzed in order to assess differences between the three methods.

### 2.5. Statistics

Statistical analyses were performed with the statistics software package JMP 11.1.1 (SAS Institute, Cary, NC, USA) and IBM SPSS Statistics 22 (IBM, Armonk, NY, USA). JMP was used to investigate the presence of normality of the data using the Shapiro-Wilk test. An ANOVA was performed to analyze differences between all three methods, when inter-device agreement was assessed. SPSS was used to calculate Intraclass Correlation Coefficients [15] and Bland Altman [16] plots were used to investigate the inter-device agreement and the differences, when refraction was assessed using the three different methods.

## 3. Results

### 3.1. Descriptive Statistics

Table 1 gives an overview on the mean refractive data (±standard deviation) of the left eye for the monocular correction of SE, J0 and J45, when refraction was assessed with all of the used methods, separated for the two examiners. Standard errors were calculated from the standard deviation divided be the square root of the sample size. The mean values and standard deviations of all three power vector components of refraction showed similar values across all three methods.

### 3.2. Bland-Altman Analysis for Inter-Device Agreement

**Figure 1:**
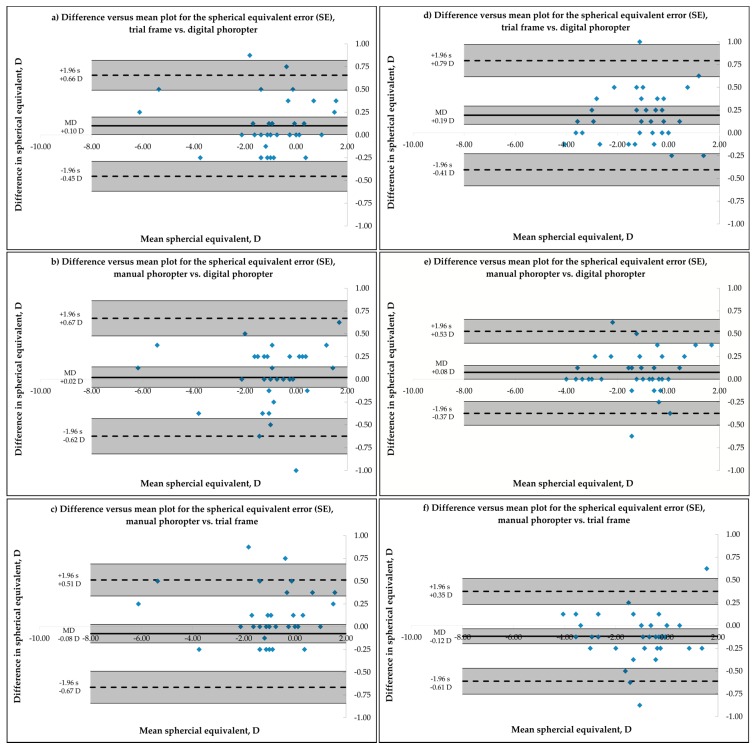
(**a**–**f**) Difference versus mean plot to compare the three subjective methods to determine the spherical equivalent refractive error (SE) of the left eye measured by examiner 1 (*n* = 36, Figure 1a–c) and 2 (*n* = 38, Figure 1d–f). (**a**) and (**d**) trial frame vs. digital phoropter; (**b**) and (**e**) manual phoropter vs. digital phoropter and (**c**) and (**f**) manual phoropter vs. trial frame. Solid line indicates the mean difference, while dashed lines represent the upper and lower limit (±95% limit of agreement). MD = mean difference and s = standard deviation. Shaded areas present 95% confidence interval limits for the mean difference and 95% limits of agreement.

Bland-Altman analysis for inter-device agreement represents the level of agreement between several measurements of the refractive error of one subject that is refracted by the same examiner under the same conditions but with different methods. Results for examiners 1 and 2 are summarized in Figure 1 for the measurement of the SE for each pair-by-pair comparison of the three methods, using a Bland-Altmann plot and 95% Limits of Agreement (LoA), calculated as 1.96 multiplied by the standard deviation of the difference [15]. Comparison of trial frame vs. digital phoropter are shown in (a) and (d), manual phoropter vs. digital phoropter in (b) and (e) and manual phoropter vs. trial frame in (c) and (f).

For examiner 1, agreement between measurements of the spherical equivalent of the refractive error and both power vector components J0 and J45 (see Table 2) was similar for all used subjective methods. A statistically significant difference was found in the measurement of the SE between the trial frame and the digital phoropter, while the trial frame showed more positive readings. Ninety-five percent LoA for the measurement of the SE was smaller between trial frame and automated phoropter for exaimer 1 (±0.56 D), followed by manual phoropter vs. trial frame (±0.59 D) and manual phoropter vs. digital phoropter (±0.65 D). Ninety-five percent CI of the lower and upper 95% LoA for the SE in case of examiner 1 were ±0.17 D (trial frame vs. digital phoropter, Figure 1a), ±0.19 D (manual phoropter vs. digital phoropter, Figure 1b) and ±0.18 D (manual phoropter vs. trial frame, Figure 1c). In the case of examiner 2, measurement of the spherical equivalent refractive error was more positive using the trial frame, when compared to either the digital (mean difference = 0.19 D, Figure 1d) or the manual phoropter (mean difference = 0.12 D, Figure 1f). When comparing the manual and the digital phoropter, the manual phoropter showed more positive measurements for the spherical equivalent (Figure 1e). Ninety-five percent LoA for the assessment of the SE was smallest for the comparison of manual vs. digital phoropter (±0.45 D), followed by the manual phoropter vs. trial frame (±0.49 D) and the trial frame vs. the digital phoropter (±0.56 D). Calculated 95% CI of the upper and lower limit of the 95% LoA for the measured SE were ±0.18 D (trial frame vs. digital phoropter, Figure 1d), ±0.13 D (manual phoropter vs. digital phoropter, Figure 1e) and ±0.10 D (manual phoropter vs. trial frame, Figure 1f). For both examiners, no influence of the refractive error of the subject’s eye on the difference between the used methods was observed. An ANOVA that analyzed the variances between the three methods for SE, J0 and J45 showed no significant differences between the methods (SE: *p* = 0.13, J0: *p* = 0.58 and J45: *p* = 0.96, two-way ANOVA) for examiner 1 and for examiner 2 (SE: *p* = 0.88, J0: *p* = 0.95 and J45: *p* = 1, two-way ANOVA).

Calculations regarding the 95% LoA were also done for both power vector components J0 and J45 to evaluate differences between the three subjective methods and the data are summarized in Table 2 for both for examiners. Additionally, the 95% CI for the upper and lower limit of the 95% LoA [15] were calculated and are presented in the same table.

### 3.3. Intra Class Correlation Analysis

In addition to the use of a Bland Altman plot and the calculation of the 95% LoA, Intra-Class Correlation coefficients (ICC) [17] were also used for the analysis of the inter-device agreement. In the conducted analysis, a two-way random absolute agreement calculation ICC(2,k) was performed and the ICCs were calculated for both examiners separately with pairwise correlations of each device and for the three power vector components of refraction (SE, J0 and J45). Results can be obtained from Table 3.

### 3.4. Time to Assess Subjective Refraction under Binocular Conditions

**Figure 2:**
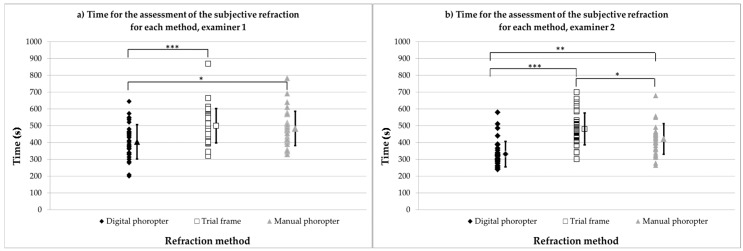
Individual and average data (±1 standard deviation) for the time of the assessment of the subjective refraction for each method, separated for examiner 1 (**a**) and examiner 2 (**b**). * = *p* < 0.05; ** = *p* < 0.01; *** = *p* < 0.001.

The time needed to assess the subjective refraction under binocular conditions was saved automatically by the i.Com software. Analysis was conducted for each measurement with all three methods and for each examiner (for examiner 1, 36; and for examiner 2, 38 measurements). Figure 2a represents the time for each subject, as well as the associated mean values ±1 standard deviations (seconds) for examiner 1, while Figure 2b represents results for examiner 2.

For both examiners, subjective refraction with the digital phoropter was significantly faster compared to the assessment when the trial frame and the manual phoropter were used.

## 4. Discussion

Many studies [8,9,10,11,12] on the reproducibility, the repeatability, and the level of agreement between different methods to measure the subjective refraction of the eye have been conducted and have resulted in different estimates for the various methods.

### 4.1. Bland-Altman Analysis for Inter-Device Agreement

For the subjective measurement of sphero-cylindrical refractive errors for repeated measures by the same examiner, previously reported 95% limit of agreement range from ±0.94 D for cycloplegic subjective refraction to ±0.63 D for the non-cycloplegic assessment of refractive errors [10]. In the case of retinoscopy, 95% limit of agreement was reported to be ±0.95 D for cycloplegic retinoscopy and ±0.78 D for non-cycloplegic retinoscopy, for repeated measures of refractive errors by the same examiner [10]. In the current study, we compared the 95% limit of agreement when refractive errors were assessed by the same examiner but with three different subjective methods for non-cycloplegic refraction in either 36 or 38 participants for examiner 1 and examiner 2, respectively. Similar 95% levels of agreement where observed, when comparing the measurement of non-cycloplegic measurement of the spherical equivalent refractive error with the three used methods for examiner 1 (95% LoA trial frame vs. digital phoropter: ±0.56 D, 95% LoA manual phoropter vs. digital phoropter: ±0.65 D, 95% LoA manual phoropter vs. trial frame: ±0.59 D) and examiner 2 (95% LoA trial frame vs. digital phoropter: ±0.60 D, 95% LoA manual phoropter vs. digital phoropter: ±0.45 D, 95% LoA manual phoropter vs. trial frame: ±0.49 D). Rosenfield and Chiu, 1995 [12] showed that the 95% limits of agreement for repeated measurements of the subjective refractive is ±0.25 D, Bullimore [18] found an 95% limit of agreement regarding the repeatability of the spherical equivalent refractive error, measured by two optometrists of ±0.78 D with a mean difference of −0.12 D.

Nevertheless, one still has to keep in mind that differences in the measurement of refractive errors would lead to different results for the correction of refractive errors. For example for examiner 2, statistically significant mean differences were found for the SE, while more positive values were measured using the trial frame (mean difference 0.19 D compared to the digital phoropter and 0.12 D compared with the manual phoropter) and the manual phoropter vs. the digital phoropter (mean difference 0.08 D). In case of examiner 1, a difference in the measurement of the SE was only observed between the trial frame and the digital phoropter (mean difference 0.10 D). As stated already in the background section, Goss and Grovenor [13] concluded that the subjective refraction is a valid and repeatable procedure. They stated that 80% of subjective refractions are within ±0.25 D and even 95% are within ±0.50 D. Compared to Goss and Grovenor, the calculated percentages of agreement for the inter-device agreement gave equal results, also when they showed differences between the individual comparisons. In case of examiner 1, the following percentages of agreement for the limits ±0.25 D and ±0.50 D regarding the measurement of the SE were observed: Trial frame vs. digital phoropter ±0.25 D: 81%/±0.50 D: 94%, manual phoropter vs. digital phoropter ±0.25 D: 69%/±0.50 D: 92% and manual phoropter vs. trial frame ±0.25 D: 78%/±0.50 D: 92%. For examiner 2, the following percentages of agreement were observed: Trial frame vs. digital phoropter ±0.25 D: 71%/±0.50 D: 92%, manual phoropter vs. digital phoropter ±0.25 D: 82%/±0.50 D: 95% and manual phoropter vs. trial frame ±0.25 D: 84%/±0.50 D: 92%. Since Rosenfield and Chiu, 1995 [12] defined that mean differences or changes in the refractive error of ±0.50 D are the minimum clinical significant shift in the refractive status, we conclude that although in case of the reported limits of agreement that were ≥±0.50 D and reported individual mean differences that showed statistical significance, the observed mean differences in the current study are minor and of clinically irrelevance. Additionally, the observed differences, especially in case of the spherical equivalent refractive error, might be influenced confounding factors, such as accommodative fluctuations and the pupil size. As discussed by Grein et al., 2014 [19], especially the fluctuations of accommodation seem to play a minor role, since the subjects normally have enough time to make their decision. One additional factor that might influence the outcome of the refractive measurement is the size of the pupil that could have been different between the different methods. Collins and colleagues measured the subjective refraction through different artificial pupils with sizes of 3, 5 and 7 mm in 10 healthy, young subjects after the pupils were dilated using phenylephrine. For these small groups of subjects, the authors reported a change of the spherical refractive error between the 3 mm and 7 mm pupil of +0.18 D (SD ± 0.24 D) [20]. Additionally, Grein and colleagues concluded that the size of the pupil only had minor influences on the measurement of refractive errors [19]. Since the lighting conditions were kept constant during the survey of the experiment, resulting most likely in pupil diameters of 4 mm or smaller the influence of different pupil sizes when measuring the refractive errors with the used methods is of minor importance for the outcome of the current study. Within the same subject and the same method, Grein and colleagues [19] reported that the 95% limit of agreement for the SE (in 24 subjects) was ±0.44 D, with a range between ±0.22 D and ±0.65 D for the individual subjects [19], indicating that also the intra-subject reliability for the measurement of their refractive error is limited to certain range and will have an influence on the inter-device agreement for the subjective measurement of refractive errors. The reported 95% LoA of the current study, as well as the percentages of agreement, reveal a good agreement between the methods and are comparable to earlier published results. Therefore, it can be concluded that all methods that were used in order to measure the subjective refraction have a precision (in terms of the the 95% LoA) small enough to measure refractive errors.

### 4.2. Intra Class Correlation Analysis

The assessment of the intra-device agreement between the measured refractive errors in both groups of subjects that were measured by the two examiner using three different subjective methods showed a good correlation for all of the three power vector components of refraction (examiner 1 trial frame vs. digital phoropter: ICC SE: 0.992; ICC J0: 0.985, ICC J45: 0.965/manual phoropter vs. digital phoropter: ICC SE 0.989; ICC J0: 0.967, ICC J45: 0.932/manual phoropter vs. trial frame: ICC SE 0.991; ICC J0: 0.974, ICC J45: 0.958; examiner 2 trial frame vs. digital phoropter: ICC SE: 0.987; ICC J0: 0.978, ICC J45: 0.953/manual phoropter vs. digital phoropter: ICC SE 0.993; ICC J0: 0.985, ICC J45: 0.963/manual phoropter vs. trial frame: ICC SE 0.991; ICC J0: 0.971, ICC J45: 0.953). Additionally, all correlations showed significance levels of *p* < 0.001. Since it was claimed that ICCs are suitable for the assessment of the agreement between two or more quantitative measures, one can conclude that the observed ICCs indicate a significant correlation (*p* < 0.001) as well as a good agreement (ICC > 0.75) between the compared subjective methods for both examiners, independent from the analyzed refractive error.

The small 95% confidence intervals for all three power vector components of the refractive errors reveals the good agreement between the tested methods. Studies regarding the ICCs for the refractive errors of the eye (sphere, spherical equivalent, cylinder, axis, J0 and J45) compared subjective refraction (mainly using trial frames) with objective refraction (using autorefractors or aberrometers) but have not compared different methods of subjective refraction so far. For example, De Carlo and colleagues [21] found high ICCs regarding the agreement for the spherical equivalent error (0.92), but not for the two astigmatic vectors J0 (0.08) and J45 (0.15) when they compared trial frame refraction and autorefraction, using the Nikon Retinomax K+ (Nikon, Inc., Tokyo, Japan) in 440 low-vision patients. The observed difference from De Carlo to the current study regarding the power vector components J0 and J45 are most likely caused by the fact that they compared trial frame refraction to autorefraction. Segura [22] investigated the repeatability of different spherocylindrical corrections in 42 eyes, obtained during a subjective refraction, with an autorefractor (WAM-5500, Shigiya Machinery Works Ltd., Fukuyama City, Hiroshima, Japan) and an aberrometer (iTrace, Tracey Technologies, Houston, TX, USA). They reported ICC’s regarding the repeatability for the spherical refraction of the autorefractor of 0.999 and 0.998 for the aberrometer. Comparing the intra-observer reliability of the SE obtained with classical trial frame refraction and autorefraction (using the WAM-5500, Grand Seiko Co. Ltd), Pujol [23] reported ICCs for the SE regarding the intra-observer reliability, when refractive errors were measured in 104 eyes of 52 subjects. In case of trial frame refraction, ICC was 0.993, while it was 0.991 for the used autorefractor (WAM-5500, Grand Seiko Co. Ltd). The reported ICCs of the current study are comparable to ICCs from previous studies investigating the intra-examiner reliability for the assessment of refractive errors.

## 5. Conclusions

The agreement of the measurement of the refractive error of the eye between different subjective methods results in comparable values to non-cylcoplegic retinoscopy or autorefraction measurements, in case of mean differences and 95% limits of agreement, as well as intra-class correlation coefficients (ICC > 0.9). All used subjective methods showed a good agreement and correlation between each other, while the refraction using the digital phoropter was significantly faster.

## Figures and Tables

**Table 1:** Mean values, ±standard deviation and ±standard error for each refractive parameter (SE, J0 and J45) for examiner 1 and 2, when refraction was assessed with all methods.

		Examiner 1 for *n* = 36 Subjects			Examiner 2 for *n* = 38 Subjects		
		Mean Value (D)	±1 Standard Deviation (D)	±1 Standard Error (D)	Mean Value (D)	±1 Standard Deviation (D)	±1 Standard Error (D)
trial frame	SE	−0.92	1.57	0.26	−1.07	1.38	0.22
	J0	0.01	0.31	0.05	0.03	0.35	0.06
	J45	0.00	0.21	0.04	0.02	0.22	0.04
manual phoropter	SE	−0.99	1.59	0.27	−1.17	1.38	0.22
	J0	0.02	0.34	0.06	0.00	0.36	0.06
	J45	−0.01	0.20	0.03	0.00	0.26	0.04
digital phoropter	SE	−1.02	1.57	0.26	−1.24	1.36	0.22
	J0	0.01	0.32	0.05	0.02	0.36	0.06
	J45	0.01	0.20	0.03	0.00	0.24	0.04

**Table 2:** Descriptive analysis for the comparison of the three subjective methods that assess the subjective refractive error, separated for the two examiners. Data represents the Bland-Altmann analysis.

**Examiner 1 (*n* = 36)**					
		**Mean Difference (D)**	**95% Limit of Agreement (D)**	**95% CI for Upper Limit (D)**	**95% CI for Lower Limit (D)**
trial frame vs. digital phoropter	SE	0.10	± 0.56	0.49 to 0.82	−0.29 to −0.62
	J0	0.00	± 0.14	0.11 to 019	−0.10 to −0.18
	J45	−0.01	± 0.14	0.09 to 0.17	−0.11 to −0.19
manual phoropter vs. digital phoropter	SE	0.06	± 0.65	0.63 to 1.01	−0.43 to −0.82
	J0	0.01	± 0.21	0.16 to 0.29	−0.14 to −0.27
	J45	−0.02	± 0.18	0.11 to 0.22	−0.14 to −0.25
manual phoropter vs. trial frame	SE	−0.04	± 0.59	0.56 to 0.91	−0.64 to −0.99
	J0	0.01	± 0.19	0.14 to 0.26	−0.12 to −0.24
	J45	−0.01	± 0.15	0.10 to 0.19	−0.11 to −0.20
**Examiner 2 (*n* = 38)**					
		**Mean Difference (D)**	**95% Limit of Agreement (D)**	**95% CI for Upper Limit (D)**	**95% CI for Lower Limit (D)**
trial frame vs. digital phoropter	SE	0.19	± 0.60	0.62 to 0.97	−0.23 to −0.58
	J0	0.00	± 0.20	0.14 to 0.25	−0.14 to −0.25
	J45	0.01	± 0.18	0.14 to 0.24	−0.11 to −0.21
manual phoropter vs. digital phoropter	SE	0.08	± 0.45	0.40 to 0.66	−0.24 to −0.51
	J0	−0.02	± 0.16	0.10 to 0.19	−0.14 to −0.23
	J45	0.00	± 0.17	0.11 to 0.22	−0.12 to −0.22
manual phoropter vs. trial frame	SE	−0.12	± 0.49	0.23 to 0.52	−0.47 to −0.75
	J0	−0.02	± 0.22	0.13 to 0.26	−0.18 to −0.30
	J45	−0.02	± 0.18	0.11 to 0.21	−0.15 to 0.25

**Table 3:** Intra-Class Correlation, their lower and upper 95% CI interval, for the pairwise correlation of each device, separated for the two examiners and for the power vector components of refraction SE, J0 and J45.

**Examiner 1 (*n* = 36)**					
		**ICC**	**95% CI**		***p***
			**Lower**	**Lower**	
trial frame vs. digital phoropter	SE	0.992	0.984	0.996	<0.01
	J0	0.985	0.97	0.992	<0.01
	J45	0.965	0.932	0.982	<0.01
manual phoropter vs. digital phoropter	SE	0.989	0.978	0.994	<0.01
	J0	0.967	0.935	0.963	<0.01
	J45	0.932	0.866	0.965	<0.01
manual phoropter vs. trial frame	SE	0.991	0.982	0.995	<0.01
	J0	0.974	0.949	0.987	<0.01
	J45	0.958	0.917	0.978	<0.01
**Examiner 2 (*n* = 38)**					
		**ICC**	**95% CI**		***p***
			**Lower**	**Lower**	
trial frame vs. digital phoropter	SE	0.987	0.975	0.993	<0.01
	J0	0.978	0.958	0.989	<0.01
	J45	0.953	0.91	0.976	<0.01
manual phoropter vs. digital phoropter	SE	0.993	0.986	0.996	<0.01
	J0	0.985	0.971	0.992	<0.01
	J45	0.963	0.928	0.981	<0.01
manual phoropter vs. trial frame	SE	0.991	0.84	0.996	<0.01
	J0	0.971	0.944	0.985	<0.01
	J45	0.953	0.909	0.975	<0.01

The intra class correlation revealed high correlations for the assessment of the refractive errors between all three used methods for both examiners. Additionally, all correlations showed high significant values (*p* < 0.001).
